# Supplementary material for: Dietary diversity is inversely correlated with pre-pregnancy body mass index among women in a Michigan pregnancy cohort
Source: PeerJ. 2018 Sep 7;6:e5526. doi: 10.7717/peerj.5526 (PMC6130235; doi:10.7717/peerj.5526)
Supplement: Supplemental Information 3 [file peerj-06-5526-s003.docx]

/** Feb 23, 2018 sub-groups compared by study**/

**PROC** **IMPORT** DATAFILE= 'c:\Sarah\Manuscripts\2017_DDW_BK\2018Feb23_DDW_data.csv' DBMS=csv

OUT=GROWTH REPLACE;

GETNAMES=YES;

GUESSINGROWS=**355**;

**RUN**;

**proc** **contents**;

**run**;

**proc** **print**;

**run**;

/**Table 1.**/

**proc** **sort** data=growth;

by BMI_cat;

**run**;

**proc** **freq** data=growth;

tables BMI_cat*study / chisq exact;

**run**;

**proc** **sort** data=growth;

by age_cat;

**run**;

**proc** **freq** data=growth;

tables age_cat*study / chisq exact;

**run**;

**proc** **sort** data=growth;

by parity_cat;

**run**;

**proc** **freq** data=growth;

tables parity_cat*study / chisq exact;

**run**;

/** Table 1. BMI categories compared by study**/

/**normal**/

**data** BMI;

input Normal $ NumYes Total;

Response="Yes"; Count=NumYes; output;

Response="No "; Count=Total-NumYes; output;

datalines;

ARCH 8 25

BABY 6 15

;

**proc** **print** noobs;

var normal Response Count;

**run**;

**proc** **freq** order=data;

weight Count;

table normal * Response / chisq exact riskdiff;

**run**;

/**overweight**/

**data** BMI;

input Normal $ NumYes Total;

Response="Yes"; Count=NumYes; output;

Response="No "; Count=Total-NumYes; output;

datalines;

ARCH 3 25

BABY 5 15

;

**proc** **print** noobs;

var normal Response Count;

**run**;

**proc** **freq** order=data;

weight Count;

table normal * Response / chisq exact riskdiff;

**run**;

/**obese**/

**data** BMI;

input Normal $ NumYes Total;

Response="Yes"; Count=NumYes; output;

Response="No "; Count=Total-NumYes; output;

datalines;

ARCH 14 25

BABY 4 15

;

**proc** **print** noobs;

var normal Response Count;

**run**;

**proc** **freq** order=data;

weight Count;

table normal * Response / chisq exact riskdiff;

**run**;

/**BMI catogories compared by study**/

/** Table 1. age groups compared by study**/

/**20to 24**/

**data** age;

input Normal $ NumYes Total;

Response="Yes"; Count=NumYes; output;

Response="No "; Count=Total-NumYes; output;

datalines;

ARCH 1 25

BABY 1 15

;

**proc** **print** noobs;

var normal Response Count;

**run**;

**proc** **freq** order=data;

weight Count;

table normal * Response / chisq exact riskdiff;

**run**;

/**25 to 29**/

**data** age;

input Normal $ NumYes Total;

Response="Yes"; Count=NumYes; output;

Response="No "; Count=Total-NumYes; output;

datalines;

ARCH 8 25

BABY 2 15

;

**proc** **print** noobs;

var normal Response Count;

**run**;

**proc** **freq** order=data;

weight Count;

table normal * Response / chisq exact riskdiff;

**run**;

/**30 to 34**/

**data** age;

input Normal $ NumYes Total;

Response="Yes"; Count=NumYes; output;

Response="No "; Count=Total-NumYes; output;

datalines;

ARCH 8 25

BABY 6 15

;

**proc** **print** noobs;

var normal Response Count;

**run**;

**proc** **freq** order=data;

weight Count;

table normal * Response / chisq exact riskdiff;

**run**;

/**35 to 39**/

**data** age;

input Normal $ NumYes Total;

Response="Yes"; Count=NumYes; output;

Response="No "; Count=Total-NumYes; output;

datalines;

ARCH 8 25

BABY 6 15

; **proc** **print** noobs;

var normal Response Count;

**run**;

**proc** **freq** order=data;

weight Count;

table normal * Response / chisq exact riskdiff;

**run**;

/**age groups compared by study**/

/**Table 1. parity compared by study**/

/**one**/

**data** parity;

input Normal $ NumYes Total;

Response="Yes"; Count=NumYes; output;

Response="No "; Count=Total-NumYes; output;

datalines;

ARCH 9 25

BABY 5 15

; **proc** **print** noobs;

var normal Response Count;

**run**;

**proc** **freq** order=data;

weight Count;

table normal * Response / chisq exact riskdiff;

**run**;

/**two**/

**data** parity;

input Normal $ NumYes Total;

Response="Yes"; Count=NumYes; output;

Response="No "; Count=Total-NumYes; output;

datalines;

ARCH 10 25

BABY 10 15

; **proc** **print** noobs;

var normal Response Count;

**run**;

**proc** **freq** order=data;

weight Count;

table normal * Response / chisq exact riskdiff;

**run**;

/** 3 or more**/

**data** parity;

input Normal $ NumYes Total;

Response="Yes"; Count=NumYes; output;

Response="No "; Count=Total-NumYes; output;

datalines;

ARCH 6 25

BABY 0 15

; **proc** **print** noobs;

var normal Response Count;

**run**;

**proc** **freq** order=data;

weight Count;

table normal * Response / chisq exact riskdiff;

**run**;

/**Parity compared by study**/

/**Fig 1. correlation between BMI and DDW score**/

**proc** **univariate** data=growth plots;

var BMI ;

by BMI_cat;

**run**;

**proc** **corr** data=growth spearman ;

var BMI MDDW_total;

**run**;

/**end**/

/**Fig 2. comparison among those achieving DDW score >5 by BMI category**/

**proc** **freq** data=growth;

tables BMI_cat*MDDW_above_5 / chisq;

**run**;

**proc** **freq** data=growth;

tables BMI_cat*MDDW_above_5 / chisq exact;

**run**;

/*end*/

/**Fig 2. comparison among those achieving DDW score >5 by BMI category normal versus obese**/

**data** meetMDDW;

input Normal $ NumYes Total;

Response="Yes"; Count=NumYes; output;

Response="No "; Count=Total-NumYes; output;

datalines;

Normal 12 14

Obese 7 18

; **proc** **print** noobs;

var normal Response Count;

**run**;

**proc** **freq** order=data;

weight Count;

table normal * Response / chisq exact riskdiff;

**run**;

/*end*/

/**fig 2. comparison among those achieving DDW score >5 by BMI category overweight versus obese**/

**data** meetMDDW;

input Normal $ NumYes Total;

Response="Yes"; Count=NumYes; output;

Response="No "; Count=Total-NumYes; output;

datalines;

Overweight 7 8

Obese 7 18

; **proc** **print** noobs;

var normal Response Count;

**run**;

**proc** **freq** order=data;

weight Count;

table normal * Response / chisq exact riskdiff;

**run**;

/*end*/

/**Fig 3. comparison among DDW scores by BMI category**/

**proc** **npar1way** data=growth wilcoxin DSCF;

var MDDW_total;

class BMI_cat;

**run**;

/*end*/

/**Figure 4 and data not shown. low nutrient density food comparisons**/

**proc** **sort** data=growth;

by BMI_cat;

**run**;

**proc** **freq** data=growth;

tables BMI_cat*savory / chisq exact;

**run**;

**proc** **freq** data=growth;

tables BMI_cat*sweets / chisq exact;

**run**;

**proc** **freq** data=growth;

tables BMI_cat*sweet_drinks / chisq exact;

**run**;

**proc** **sort** data=growth;

by study;

**run**;

**proc** **freq** data=growth;

tables study*savory / chisq exact;

**run**;

**proc** **freq** data=growth;

tables study*sweets / chisq exact;

**run**;

**proc** **freq** data=growth;

tables study*sweet_drinks / chisq exact;

**run**;

/**end low nutrient density foods**/

/**Table 2. MDDW categories by BMI categories and if they met minimum dietary diversity**/

**proc** **freq** data=growth;

tables BMI_cat*MDDW_above_5*MDDW_grains;

**run**;

**proc** **freq** data=growth;

tables MDDW_above_5*MDDW_grains / chisq exact;

**run**;

**proc** **freq** data=growth;

tables BMI_cat*MDDW_above_5*MDDW_pulses;

**run**;

**proc** **freq** data=growth;

tables MDDW_above_5*MDDW_pulses/ chisq exact;

**run**;

**proc** **freq** data=growth;

tables BMI_cat*MDDW_above_5*MDDW_nuts;

**run**;

**proc** **freq** data=growth;

tables MDDW_above_5*MDDW_nuts/ chisq exact;

**run**;

**proc** **freq** data=growth;

tables BMI_cat*MDDW_above_5*MDDW_dairy;

**run**;

**proc** **freq** data=growth;

tables MDDW_above_5*MDDW_dairy/ chisq exact;

**run**;

**proc** **freq** data=growth;

tables BMI_cat*MDDW_above_5*MDDW_meat;

**run**;

**proc** **freq** data=growth;

tables MDDW_above_5*MDDW_meat/ chisq exact;

**run**;

**proc** **freq** data=growth;

tables BMI_cat*MDDW_above_5*MDDW_eggs;

**run**;

**proc** **freq** data=growth;

tables MDDW_above_5*MDDW_eggs/ chisq exact;

**run**;

**proc** **freq** data=growth;

tables BMI_cat*MDDW_above_5*MDDW_darkGreen;

**run**;

**proc** **freq** data=growth;

tables MDDW_above_5*MDDW_darkgreen/ chisq exact;

**run**;

**proc** **freq** data=growth;

tables BMI_cat*MDDW_above_5*MDDW_vitARich;

**run**;

**proc** **freq** data=growth;

tables MDDW_above_5*MDDW_vitArich/ chisq exact;

**run**;

**proc** **freq** data=growth;

tables BMI_cat*MDDW_above_5*MDDW_veggies;

**run**;

**proc** **freq** data=growth;

tables MDDW_above_5*MDDW_veggies/ chisq exact;

**run**;

**proc** **freq** data=growth;

tables BMI_cat*MDDW_above_5*MDDW_fruit;

**run**;

**proc** **freq** data=growth;

tables MDDW_above_5*MDDW_fruit/ chisq exact;

**run**;
